# Elevated Methylation Contributes to Suppressed Expression of Special AT‐Rich Sequence‐Binding Protein 2 in Colorectal Cancer: A Gene‐Disease Association Study

**DOI:** 10.1002/hsr2.71056

**Published:** 2025-07-09

**Authors:** Weitong Cui, Cong Lu, Huaru Xue, Lei Wei, Shuai Li, Lianzheng Su, Dianfang Wei, Xiaoyu Feng, Kai Wang, Chao Song

**Affiliations:** ^1^ School of Basic Medicine Qilu Medical University Zibo Shandong Province China; ^2^ Department of Hematology Zibo Central Hospital Zibo Shandong Province China; ^3^ School of Medical Imaging Qilu Medical University Zibo Shandong Province China

**Keywords:** colorectal cancer, expression, methylation, promoter, SATB2

## Abstract

**Background and Aims:**

Special AT‐rich sequence‐binding protein 2 (SATB2) is a commonly used clinical marker for colorectal cancer (CRC) diagnosis. It has been reported that SATB2 expression is significantly downregulated in CRC tissues; however, the underlying mechanism remains unclear. Methylation represents one of the key epigenetic modifications involved in regulating gene expression. The objective of this study was to investigate the relationship between the methylation status of SATB2 and its expression levels in CRC tissues.

**Methods:**

We first investigated the methylation status and expression level of SATB2 via the use of transcriptional and methylation data from CRC patients in The Cancer Genome Atlas via bioinformatics analysis. Next, we explored the methylation status of the SATB2 promoter and SATB2 expression patterns in the collected CRC tumor and adjacent normal tissues, as well as different cell lines, via methylation‐specific PCR and quantitative PCR. Next, the normal colorectal FHC cell line and CRC cell lines were treated with azacytidine (AZA) to investigate whether the mRNA expression of SATB2 could be restored by demethylating agents. Finally, associations between clinicopathological characteristics and SATB2 methylation status were analyzed.

**Results:**

The results of both bioinformatics and experimental analyses demonstrated that SATB2 expression was downregulated in CRC tumor tissues and that SATB2 hypermethylation was detected in various regions, including the promoter. The downregulated expression of SATB2 in CRC tumor tissues was significantly correlated with SATB2 hypermethylation, and the mRNA expression level of SATB2 significantly increased following AZA treatment in certain CRC cell lines. Additionally, the methylation state of SATB2 was correlated with tumor differentiation and metastasis.

**Conclusion:**

In summary, increased methylation levels contribute to decreased SATB2 expression in CRC, indicating the clinical relevance of SATB2 methylation as a potential therapeutic target for CRC.

## 1 Introduction

Colorectal cancer (CRC) is a multifactorial disease that poses a public health concern [1]. In America, CRC is now the leading cause of cancer death in men and the second in women [2]. In China, CRC incidence in the whole population has been increasing in recent years, similar to trends in the United States [3]. Although significant advancements have been achieved in identifying prognostic markers and developing treatment strategies for CRC, the exact mechanisms of tumorigenesis underlying CRC remain to be fully elucidated. Epigenetics, characterized by changes in organisms that arise from modifications in gene expression rather than alterations to the genetic code itself, plays a central role in CRC pathogenesis [4]. Epigenetic modifications commonly observed in CRC include DNA methylation, histone modification, and noncoding RNA [5, 6, 7, 8, 9].

SATB2 serves as a highly sensitive and specific biomarker for CRC diagnosis [10, 11]. As a chromatin organizer, SATB2 is highly expressed in normal colorectal tissues; however, it has been reported that SATB2 is downregulated in a subset of CRC patients [10, 12]. Downregulation of SATB2 expression in CRC tissue has been found to be associated with altered expression levels of various microRNAs (miRNAs), such as miR‐31, miR‐182, miR‐34c‐5p, and miR‐449a [12, 13, 14, 15]. DNA methylation is also an important epigenetic modification responsible for transcriptional silencing and has been found to play an important role in cancer development [16, 17]. However, the correlation between the methylation status and the expression level of SATB2 in CRC remains largely unknown.

In this study, we first investigated the methylation status and expression patterns of SATB2 by exploring the mRNA expression and methylation data of CRC in The Cancer Genome Atlas (TCGA). We subsequently studied the methylation status of SATB2 in the promotor region and the correlation between SATB2 methylation and SATB2 expression in collected CRC tissues and adjacent normal tissues. We observed that SATB2 exhibited a greater frequency of methylation in CRC tissues than in noncancerous tissues. Additionally, we demonstrated that treatment with the demethylating agent azacytidine (AZA) restored SATB2 mRNA expression in a subset of colorectal cancer cell lines. In light of these findings, SATB2 may represent a promising therapeutic target for CRC. This study deepens our understanding of the epigenetic regulation of SATB2 in the pathogenesis of CRC and holds significant implications for CRC treatment.

## 2 Materials and Methods

### 2.1 Acquisition of mRNA Expression, Gene Methylation, and Clinical Data From TCGA

The raw RNA Sequencing (RNA‐Seq) read count data, gene methylation data, and clinical data of all available tumor tissues of colon adenocarcinoma (COAD) and rectum adenocarcinoma (READ), as well as adjacent normal tissues, were sourced from TCGA. Only primary solid tumor tissue samples and corresponding solid normal tissue samples were included to maintain sample consistency. If a sample was sequenced multiple times, the duplicate data were excluded. After the exclusion process, the RNA‐Seq datasets for COAD comprised 456 tumor samples and 41 adjacent normal samples, respectively, and the numbers were 166 and 10, respectively, for READ. The methylation datasets of COAD included 307 CRC tumor samples and 38 adjacent normal samples, while the numbers were 98 and 7, respectively, for READ.

### 2.2 Human Tissue Samples and Cell Lines

Patients with CRC were confirmed according to the morphological criteria of the World Health Organization. The inclusion criteria were as follows: (I) pathologically diagnosed with CRC; (II) willing to provide consent for participation in the study; and (III) aged between 18 and 80 years. The exclusion criteria were as follows: (I) incomplete clinicopathological data; (II) a history of other malignancies, or with additional genetic disorders; and (III) preoperative chemotherapy, radiotherapy, or immunotherapy. A total of 62 paraffin‐embedded CRC samples that met the inclusion and exclusion criteria were obtained from the Department of Pathology of Zibo Central Hospital between 2020 and 2023. Paired fresh CRC tissues and adjacent normal colorectal tissues from 28 patients were collected during surgical resection for the purpose of quantifying SATB2 mRNA expression. The study was approved by the Medical Ethics Committee and Internal Review Boards of Zibo Central Hospital (No. YXLL2022006) on March 8, 2022. Preoperatively, informed consent for the use of histopathological samples and clinical data for research purposes was obtained from all patients. Additionally, patients were assured of the opportunity to decline participation in research through an opt‐out mechanism. The utilization of human samples adhered to all pertinent national regulations and the principles outlined in the Helsinki Declaration.

The normal colorectal cell line (FHC) and eight CRC cell lines (HCT116, HT29, SW620, SW480, CACO2, LOVO, DLD‐1, and RKO) were obtained from Wuhan Pricella Biotechnology Co. Ltd, China and cultured with 5% CO_2_ at 37°C. All the cell lines were cultured in DMEM medium supplemented with 10% fetal bovine serum.

### 2.3 Methylation‐Specific PCR (MSP)

Genomic DNA was extracted from both formalin‐fixed paraffin‐embedded tissues and fresh‐frozen tissues using the TIANamp Genomic DNA Kit (Tiangen). The unmethylated cytosines present in the extracted DNA were subsequently converted to uracils using the EpiTect Bisulfite Kit (Qiagen). The MethPrimer was used for designing the MSP primers [18]. The primer sequences for SATB2 hypermethylation were as follows: forward, 5′‐TCGGGTGGTATAATTTTTTTTAGTC‐3′ and reverse, 5′‐ACACTTAATTTACAAAACCGACGTT‐3′. The primer sequences for SATB2 hypomethylation were as follows: forward, 5′‐TGGGTGGTATAATTTTTTTTAGTTGT‐3′ and reverse, 5′‐ACACTTAATTTACAAAACCAACATT‐3′. The amplified MSP products were subjected to gel electrophoresis and visualized with a KETA G Imaging System (WEALTEC).

### 2.4 Quantitative PCR (QPCR) Analysis

Total RNA from CRC tissues and cell lines was isolated using TRIzol reagent (Invitrogen), and the RevertAid First Strand cDNA Synthesis Kit (Thermo Fisher) was used to synthesize the first‐strand cDNA. Both steps were performed according to the manufacturers' instructions. The samples were subjected to 25‐μL reactions using the UltraSYBR Green Master Kit (CWBIO) on a Pangaea Real Time PCR System (Aperbio). The temperature procedure was set as follows: 95°C for 10 min followed by 40 cycles of 95°C for 15 s and 60°C for 1 min. The primers for the qPCR were as follows: SATB2 forward, 5′‐ CAAGAGTGGCATTCAACCGCAC ‐3′ and SATB2 reverse, 5′‐ ATCTCGCTCCACTTCTGGCAGA ‐3′ (OriGene, USA); GAPDH forward, 5′‐ TCTGACTTCAACAGCGACAC‐3′ and GAPDH reverse, 5′‐ CAAATTCGTTGTCATACCAG ‐3′ (Sangon Biotech). The mRNA expression level of SATB2, normalized to that of GAPDH, was determined via the ΔΔCT method.

### 2.5 AZA Treatment and Expression Change Analysis

The cells were inoculated in cell culture dishes (90 mm × 15 mm) at 3 × 10^5 ^cells/dish. When the degree of fusion reached approximately 50%, the culture medium was replaced with fresh culture medium containing AZA with the final concentration of 5 µM. The control group was supplemented with an equivalent volume of DMSO. Both the control and treatment groups included five replicates. The culture media containing AZA were refreshed every 24 h. The cells were treated with AZA or DMSO for 48 h, after which they were collected via cell scrapers. Following total RNA extraction, the expression levels of SATB2 in both the AZA treatment and control groups were assessed via qPCR. Moreover, the methylation status of SATB2 after AZA treatment was analyzed as described above.

### 2.6 Immunohistochemistry (IHC)

For histologic analysis, paraffin‐embedded tissue sections were initially rehydrated, followed by antigen retrieval utilizing sodium citrate buffer (pH 6). The SP Kit (Broad Spectrum) (catalog number: SP0041), which consists of goat sera (blocking reagent), H_2_O_2_, biotinylated sheep anti mouse/rabbit IgG (secondary antibody), streptavidin‐HRP, diaminobenzidine (DAB) substrate, and hematoxylin, was obtained from Solarbio (Beijing) for immunohistochemical staining and detection. Endogenous peroxidase activity was inhibited by treatment with 3% H_2_O_2_ for 10 min. After been blocked with goat serum, the sections were incubated with the primary antibody anti‐SATB2 (catalog number: ab34735; Abcam) overnight. The sections were incubated with a biotinylated secondary antibody for 60 min. Next, streptavidin‐HRP was added to the slides for a 10‐min incubation. The DAB substrate was subsequently added and incubated until optimal coloration was attained. Finally, the slides were subjected to counterstaining with hematoxylin. The slides were washed with PBS for three times between each step. Images were obtained via a microscope (BX51, Olympus). The staining intensity was scored by two independent observers in the same way as Gu et al. [12].

### 2.7 Statistical Analysis

Statistical analyses and plotting were performed with R (v 4.0.2). The *edgeR* package (v 3.30.3) was used for the comparative analysis of RNA‐Seq data obtained from TCGA. Boxplots showing the distribution of the expression or methylation of SATB2 were generated via *ggpubr* (v 0.4.0). The differences in the mRNA expression and methylation levels of SATB2 between tumor samples and normal samples were analyzed with two‐sided Wilcoxon tests. Pearson correlation analysis was performed to investigate the correlation between the mRNA expression and methylation levels of SATB2. The chi‐square test was used to assess the associations between SATB2 methylation and clinicopathological parameters, as well as the correlations between SATB2 methylation and mRNA expression levels. If expected counts in any cell in the contingency table were less than 5, the chi‐square test was performed with Yates' continuity correction. The significance level was set at *p* < 0.05 for all the statistical analyses.

## 3 Results

### 3.1 Expression Levels of SATB2 in TCGA Cohort on the Basis of RNA‐Seq Data for CRC

**FIGURE 1:**
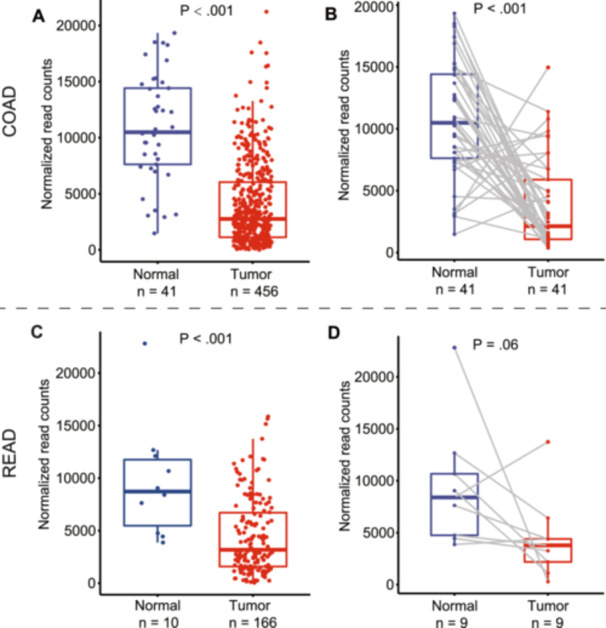
The mRNA expression levels of SATB2 in tumor and normal tissue samples of COAD (A) and READ (C) from TCGA. The expression data between matched pairs of tumor and normal tissues are show on the right (B and D). COAD, colon adenocarcinoma; READ, rectum adenocarcinoma; TCGA, The Cancer Genome Atlas.

In TCGA cohort, CRC is divided into two types: COAD and READ. As shown in Figure 1A,C, the mRNA expression level of SATB2 in tumor tissues from both COAD and READ patients was significantly lower than that in adjacent normal tissues, consistent with the results of previous studies [10, 12, 19]. The difference in mRNA expression levels between paired CRC tumor and normal tissues (Figure 1B,D) was similar to that obtained from all samples, but the difference was nonsignificant for READ (*p* = 0.06), probably due to the small sample size of the READ data set and severe sample heterogeneity [20, 21].

### 3.2 Methylation Status of SATB2 in TCGA Cohort Based on Colorectal Data

**FIGURE 2:**
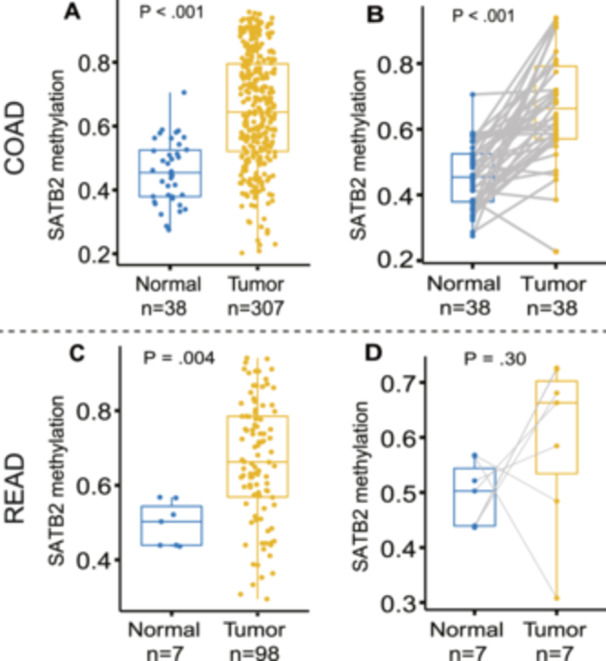
Methylation levels of cg18258980 in SATB2 in tumor and normal tissue samples of COAD (A) and READ (C) from TCGA. Methylation data between matched pairs of tumor and normal tissues are show on the right (B and D). COAD, colon adenocarcinoma; READ, rectum adenocarcinoma; TCGA, The Cancer Genome Atlas.

To investigate whether SATB2 is hypermethylated in CRC tumor tissues, we used MethSurv [22], which contains the methylation information of 35 CpG sites for SATB2, to explore the methylation data of CRC in TCGA. By analyzing the differences in the methylation status of these CpG sites between CRC tissues and adjacent normal tissues, we identified a hypermethylated CpG site in CRC tissues, namely, cg18258980, which is located in the open sea region (areas of the genome that are located more than 4 kb from CpG islands) of SATB2. As shown in Figure 2A,C, the methylation level of cg18258980 in tumor tissues from both COAD and READ patients was significantly greater than that in normal samples. Although the difference in the methylation level of cg18258980 between paired tumor and normal samples from patients with COAD (Figure 2B) was consistent with the results obtained for all samples, the difference was not significant for paired samples from patients with READ (Figure 2D), which may also be attributed to the small sample size.

### 3.3 Negative Correlation Between the Methylation and mRNA Expression of SATB2 in CRC Tissue

**FIGURE 3:**
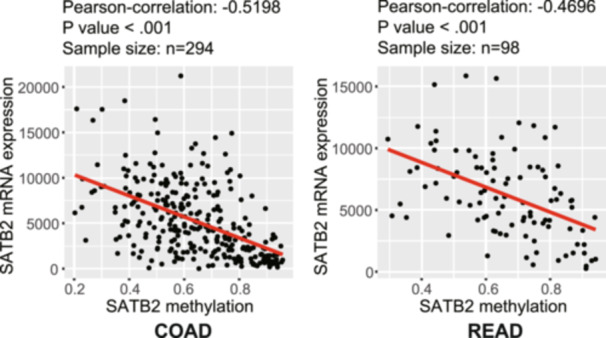
Correlation between the methylation and mRNA expression of SATB2 in the TCGA cohort on the basis of colorectal data. TCGA, The Cancer Genome Atlas.

To explore the correlation between the methylation level of cg18258980 and the mRNA expression of SATB2, we performed a correlation analysis using the methylation and RNA‐seq data of SATB2 in TCGA. As shown in Figure 3, in both COAD and READ, the methylation level of cg18258980 exhibited a significant negative correlation with the mRNA expression level of SATB2, indicating that the hypermethylation of cg18258980 in SATB2 inhibited its mRNA expression.

### 3.4 Correlation between MRNA Expression and Promoter Region Hypermethylation of SATB2 in CRC

**FIGURE 4:**
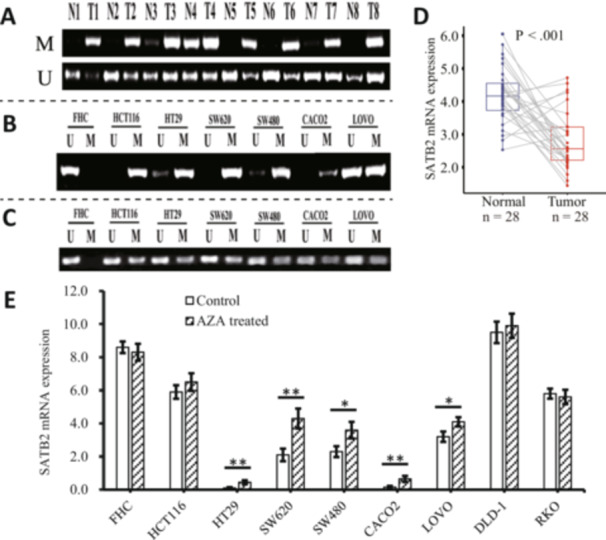
Promoter methylation is correlated with reduced mRNA expression of special AT‐rich sequence‐binding protein 2 (SATB2). (A) MSP assay results showing the methylation status of SATB2 in representative colorectal cancer (CRC) tissues (T) and matched adjacent normal colorectal tissues (N). (B, C) MSP assay results showing the methylation state of SATB2 in the normal colorectal FHC cell line and in the cancerous CRC cell lines before (B) and after (C) azacytidine (AZA) treatment. (D) qPCR results showing the mRNA expression levels of SATB2 in 28 pairs of CRC tumor and matched adjacent normal tissues. (E) Changes in SATB2 mRNA expression in FHC and CRC cell lines after AZA treatment, as determined by qPCR. M, methylated primer. U, unmethylated primer. Error bars, SD. *p < 0.05. **p < 0.01.

Hypermethylation within the promoter region is an important epigenetic mechanism affecting gene expression, but no hypermethylated site in the promoter region of SATB2 was found via the methylation data of CRC in TCGA. To explore whether the promoter region of SATB2 was hypermethylated in CRC tumor tissues, we first predicted the CpG island in the promoter region of SATB2 and designed MSP primers via MethPrimer (Supporting Information S1: Figure 1). Then, MSP was performed to examine the methylation pattern of SATB2 in the promoter region in both CRC tumor and adjacent normal tissues. As shown in Figure 4A, significantly greater hypermethylation of the SATB2 promoter was detected in CRC tissues (43/62, 69.4%) than in normal colorectal tissues (15/62, 24.2%) (Supporting Information S1: Table 1). Consistently, a hypermethylated SATB2 promoter was detected in six CRC cell lines, but not in normal colorectal FHC cell line (Figure 4B). After AZA treatment, the intensity of hypermethylation in most tumor cell lines decreased significantly, while the intensity of hypomethylation reappeared or increased significantly (Figure 4C).

QPCR was used to quantify the mRNA expression of SATB2 in paired fresh CRC and normal tissue samples. As presented in Figure 4D, a significant decrease in SATB2 mRNA expression was observed in CRC tissues compared with normal tissues, which is in line with the results obtained via TCGA data. Figure 4D further revealed that the mRNA expression levels of SATB2 were increased in 7 paired tumor‐normal CRC tissues, diverging from the expression pattern noted in the other 21 pairs. An assessment of the methylation status of SATB2 promoter in the 28 CRC tissue samples revealed that 17 out of the 21 samples whose mRNA expression was downregulated presented a hypermethylated SATB2 promoter, whereas the remaining seven samples whose mRNA expression was upregulated presented only two samples with a hypermethylated SATB2 promoter (Table 1), indicating that the methylation status at the promoter region of SATB2 was also correlated with its mRNA expression level.

**TABLE 1:** Correlation between SATB2 promoter methylation status and changes in mRNA expression in 28 CRC tissues.

Group	*n*	SATB2 promoter		*p*	Odds ratio
		Hypermethylated	Hypomethylated		
*SATB2*high	21	17 (81.0%)	4 (19.0%)	0.02	9.50
*SATB2*low	7	2 (28.6%)	5 (71.4%)		

Abbreviation: CRC, colorectal cancer; SATB2, special AT‐rich sequence‐binding protein 2.

To further elucidate the relationship between the methylation and expression levels of SATB2, we treated FHC and eight CRC cell lines with AZA to assess whether reduced methylation levels influence SATB2 expression. Following AZA treatment, significantly increased SATB2 mRNA expression was detected in the HT29, SW620, SW480, CACO2, and LOVO cell lines (Figure 4E). AZA treatment had little effect on the mRNA expression of SATB2 in the HCT116, DLD‐1, and RKO cell lines, which are known to express SATB2 [23]. These findings suggest that the low expression of SATB2 observed in certain CRC cell lines can be partially attributed to its hypermethylation.

### 3.5 Protein Expression of SATB2 in CRC

**FIGURE 5:**
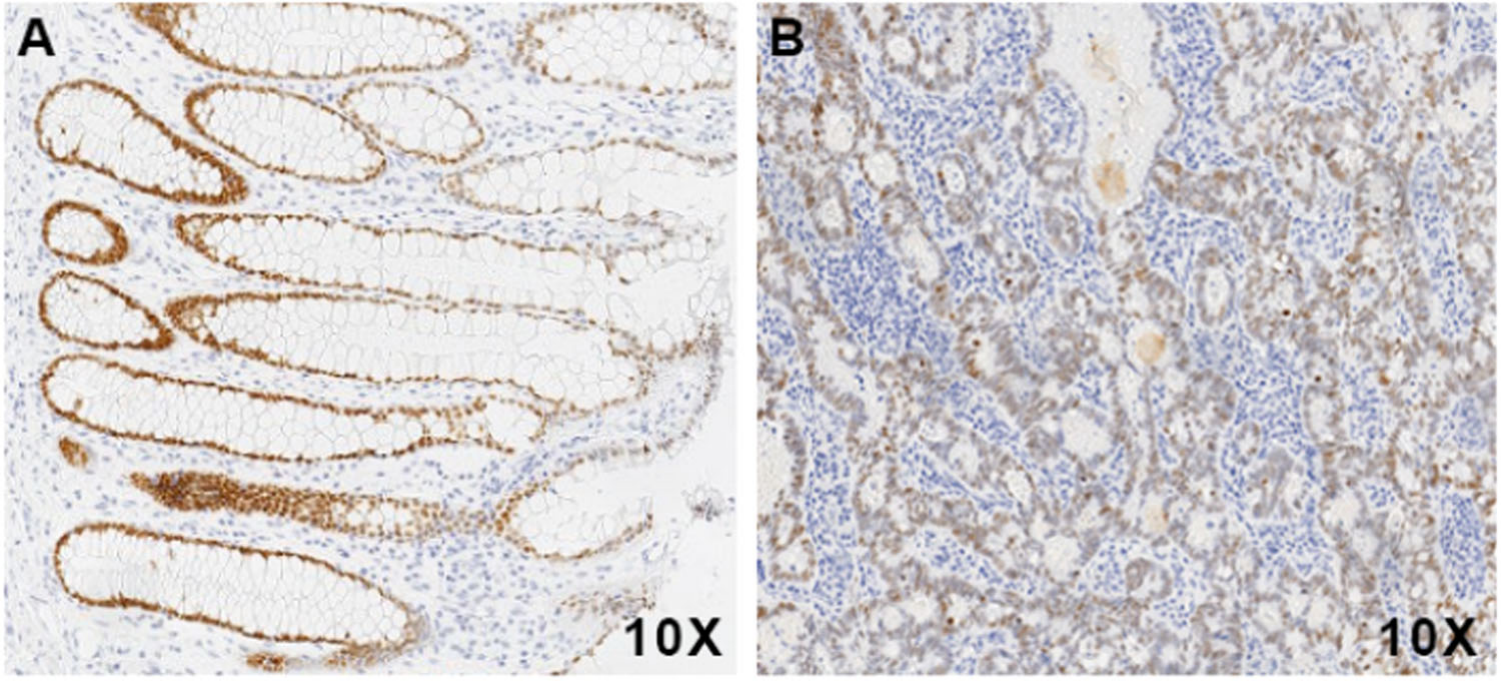
Representative immunohistochemical staining results of SATB2 in normal colorectal tissue (A) and CRC tissue (B). CRC, colorectal cancer; SATB2, special AT‐rich sequence‐binding protein 2.

The protein expression of SATB2 in CRC has been investigated via different methods in several studies [10, 11, 12, 19, 24]. To confirm the expression status of SATB2 in CRC, IHC was carried out on 62 paired CRC and adjacent normal tissues. The IHC results revealed that the positive SATB2 staining rates for the 62 primary CRC tumor and adjacent normal tissues were 95.16% (59/62) and 98.39% (61/62), respectively. Although most CRC tumor and adjacent normal tissues were positive for SATB2 expression, the protein expression level of SATB2 in most CRC tumor tissue samples was lower than that in their normal counterparts (Figure 5), which was consistent with the mRNA expression pattern of SATB2. IHC results also revealed that well‐differentiated CRC tissues presented more intense SATB2 staining than poorly differentiated CRC tissues did, which is consistent with the findings of a previous study [25].

### 3.6 Relationships Between Clinicopathological Characteristics and the Methylation Status of SATB2

No significant correlation was found between the methylation status of the SATB2 promoter and clinical characteristics, such as age, gender, tumor size, or tumor location (Table 2). Nevertheless, the methylation status of the SATB2 promoter was significantly correlated with tumor differentiation (*p* = 0.04) and lymphatic metastasis (*p* = 0.02) in CRC patients (Table 2).

**TABLE 2:** Associations between clinicopathological features and SATB2 promoter methylation status in 62 CRC patients.

Variables	*n*	SATB2 promoter		*χ*²	*p*
		Hypermethylated	Hypomethylated		
Age (year)				1.287	0.26
60	36	27	9		
≤ 60	26	16	10		
Gender				0.054	0.82
Male	34	24	10		
Female	28	19	9		
Tumor size (cm)				2.541	0.11
5	29	23	6		
≤ 5	33	20	13		
Tumor differentiation				4.228	0.04
Well and moderate	37	22	15		
Poor	25	21	4		
Tumor location				0.107	0.74
Right	13	10	3		
Left	49	33	16		
Lymphatic metastasis				5.078	0.02
Yes	39	31	8		
Not	23	12	11		

Abbreviation: CRC, colorectal cancer; SATB2, special AT‐rich sequence‐binding protein 2.

## 4 Discussion

In normal adult tissues, SATB2 displays a tissue‐specific expression pattern, with intense nuclear localization observed in nearly all glandular epithelial cells of the lower gastrointestinal tract, including the appendix, colon, and rectum [26]. Through transcriptomic and immunohistochemical analyses, SATB2 was also detected in CRC tissues, although its expression levels were markedly lower than those in normal tissues, which suggested that SATB2 might function as a tumor suppressor in CRC. Additionally, our findings revealed that the mRNA expression level of SATB2 in CRC tissue was negatively correlated with its methylation level. Encouragingly, AZA administration inhibited the hypermethylation identified in certain CRC cell lines, thereby restoring SATB2 mRNA expression.

Human CRC tissues often exhibit various epigenetic changes, such as global DNA hypomethylation, promoter region hypermethylation, histone modifications, and aberrant miRNA expression patterns [7, 8, 9]. Lee et al. reported that the loss of SATB2 in some subsets of CRCs was correlated with a high CpG island methylator phenotype [27]. In the present study, we found that SATB2 was hypermethylated at both promoter and non‐promoter regions.

The methylation of CpG sites within promoter regions is crucial for gene regulation, especially in the context of gene silencing [28]. In addition to being methylated in the promoter region, SATB2 is also hypermethylated at the CpG site cg18258980, which is located in the region of the open sea. A previous study revealed that hypomethylated regions are often situated in genomic open sea regions and are associated with chromosomal instability, gene activation, and loss of imprinting [29]. It is worth emphasizing that, beyond alterations at cg18258980, methylation alterations at other CpG sites of SATB2 may also modulate its expression.

Although a negative correlation was found between the hypermethylation of SATB2 and its mRNA expression, this did not mean that the downregulated expression of SATB2 in CRC tumor tissue was caused entirely by hepermethylation. In addition to a high CpG island methylator phenotype, factors such as high microsatellite instability, BRAF/RNF43 mutations, consensus molecular subtypes, and high tumor mutational burden are also correlated with decreased mRNA expression of SATB2 [27]. Several specific miRNAs, such as miR‐31, miR‐34c‐5p, miR‐182, and miR‐449a, play a role in the regulation of SATB2 expression in CRC [12, 13, 14, 15]. SATB2‐AS1, a long noncoding RNA derived from the antisense strand of SATB2, also plays a role in modulating SATB2 expression [30]. Moreover, a recent study demonstrated that histone methylation influences the expression of SATB2 in neuronal cells [31]. However, it remains unclear whether the expression of SATB2 in CRC tissues is regulated by histone modifications. Therefore, the expression of SATB2 is under complex regulation by multiple epigenetic factors.

Many studies have concluded that the expression level of SATB2 is associated with CRC prognosis. Wang et al. [32] found that under‐expression of SATB2 was strongly associated with poor prognosis, tumor invasion, lymph node metastasis, distant metastasis, and Dukes' classification in CRC. Eberhard et al. [33] reported that elevated SATB2 expression was correlated with prolonged cancer‐specific survival and overall survival in CRC patients, serving as an independent marker of good prognosis in patients with colon cancer but not in those with rectal cancer, similar to the findings of Mezheyeuski et al. [34]. Furthermore, multiple studies have confirmed the link between reduced SATB2 expression and an inferior overall prognosis [12, 24, 35, 36]. Our findings demonstrated that hypermethylation of SATB2 was highly correlated with poor differentiation and lymphatic metastasis in CRC; therefore, the methylation level of SATB2 may serve as a prognostic marker for CRC.

There are several limitations in our research. First, the TCGA database contains only seven adjacent normal tissue samples from READ, which may compromise its statistical power. Second, although the differences in methylation levels at other CpG sites between COAD and READ were inconsistent or not statistically significant, these changes may still affect SATB2 expression. Furthermore, in addition to methylation, gene expression is intricately regulated by various factors, such as histone modifications and noncoding RNAs. Although we identified a negative correlation between reduced expression and elevated methylation of SATB2 in CRC, further efforts are necessary to comprehensively elucidate the regulatory mechanisms governing SATB2 expression.

## 5 Conclusions

SATB2 is significantly hypermethylated in the promoter and open sea area in CRC. Hypermethylation of SATB2 is highly correlated with its downregulated expression, as well as poor differentiation and lymphatic metastasis. Given that hypermethylation of SATB2 suppresses its mRNA expression in CRC tissues and that AZA effectively enhances SATB2 expression in certain CRC cell lines, SATB2 has the potential to be a therapeutic target for particular CRC patients. Although hypermethylation of SATB2 has been confirmed in CRC tissue, the mechanisms of SATB2 hypermethylation and the effects of SATB2 hypermethylation on the expression of its target genes remain to be further studied.

## Author Contributions

**Weitong Cui:** writing – original draft, formal analysis, methodology, investigation, funding acquisition, software. **Cong Lu:** formal analysis, writing – original draft, methodology, investigation, data curation, validation; resources. **Huaru Xue:** investigation, visualization. **Lei Wei:** writing – review and editing. **Shuai Li:** formal analysis, data curation. **Lianzheng Su:** formal analysis. **Dianfang Wei:** writing – review and editing, formal analysis. **Xiaoyu Feng:** writing – review and editing, formal analysis. **Kai Wang:** conceptualization, writing – review and editing, project administration, supervision. **Chao Song:** data curation, conceptualization, writing – review and editing, project administration, funding acquisition, resources.

## Ethics Statement

The study was approved by the Medical Ethics Committee and Internal Review Boards of Zibo Central Hospital (No. YXLL2022006) on March 8, 2022, and performed in accordance with the Declaration of Helsinki and in compliance with relevant laws and guidelines in China.

## Conflicts of Interest

The authors declare no conflicts of interest.

## Transparency Statement

The lead author Kai Wang, Chao Song affirms that this manuscript is an honest, accurate, and transparent account of the study being reported; that no important aspects of the study have been omitted; and that any discrepancies from the study as planned (and, if relevant, registered) have been explained.

## Supporting information

Supporting Information.

## Data Availability

The methylation and mRNA expression data that support the findings of this study are openly available in TCGA (https://www.cancer.gov/tcga) and GTEx (https://www.cancer.gov/tcga). The pathological and clinical data of the patients, securely stored at Zibo Central Hospital, are available from the corresponding author upon reasonable request.
