# Supplementary material for: Elevated Methylation Contributes to Suppressed Expression of Special AT‐Rich Sequence‐Binding Protein 2 in Colorectal Cancer: A Gene‐Disease Association Study
Source: Health Sci Rep. 2025 Jul 9;8(7):e71056. doi: 10.1002/hsr2.71056 (PMC12239154; doi:10.1002/hsr2.71056)
Supplement: Supplementary file 1 — Supporting Information. [file HSR2-8-e71056-s001.docx]

**Supporting Information**

**Figure 1**


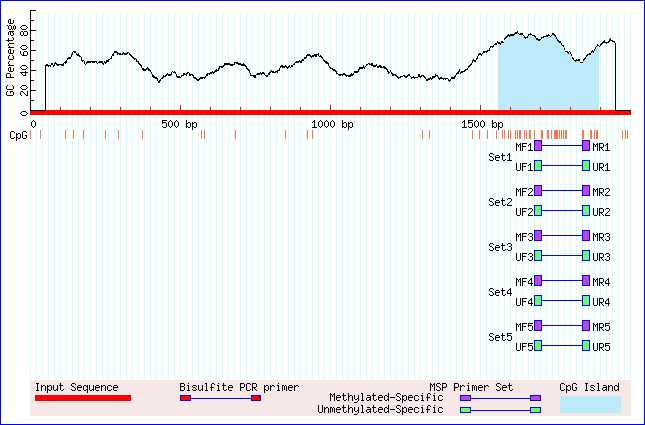


**Figure 1.** The CpG island in the promoter region of SATB2 and correspondent MSP primers predicted and designed using MethPrimer.

**Table 1.** Methylation status of SATB2 promoter in CRC and adjacent normal tissues.

| Group | n | SATB2 promoter | | *P* | χ² |
| --- | --- | --- | --- | --- | --- |
|  |  | Hypermethylated | Hypomethylated |  |  |
| CRC tissue | 62 | 43 (69.4 %) | 19 (30.6 %) | < 0.001 | 25.396 |
| Adjacent normal tissue | 62 | 15 (24.2 %) | 47 (75.8 %) |  |  |
